# Differential response of IgM and IgG memory B cell populations to CD40L: insights of T cell – memory B cell interactions

**DOI:** 10.3389/fimmu.2024.1432045

**Published:** 2024-07-10

**Authors:** Hector Rincon-Arevalo, Ana-Luisa Stefanski, Tuan Anh Le, Marcos Cases, Annika Wiedemann, Franziska Szelinski, Jacob Ritter, Van Duc Dang, Andreia C. Lino, Thomas Dörner, Eva Schrezenmeier

**Affiliations:** ^1^ Department of Medicine/Rheumatology and Clinical Immunology, Charité-Universitätsmedizin Berlin, Berlin, Germany; ^2^ Department of Medicine/Nephrology and Medical Intensive Care, Charité-Universitätsmedizin Berlin, Berlin, Germany; ^3^ Deutsches Rheuma-Forschungszentrum Berlin, ein Institut der Leibniz Gemeinschaft, Berlin, Germany; ^4^ Grupo de Inmunología Celular e Inmunogenética, Facultad de Medicina, Instituto de Investigaciones Médicas, Universidad de Antioquia UdeA, Medellín, Colombia

**Keywords:** memory B cell, plasmablast, CD40L, plasma cell, T:B co-stimulation

## Abstract

Memory B cells (mBCs) are characterized by their long-term stability, fast reactivation, and capability to rapidly differentiate into antibody-secreting cells (ASCs). However, the role of T cells in the differentiation of mBCs, in contrast to naive B cells, remains to be delineated. We study the role of T cells in mBC responses, using CD40L stimulation and autologous T-B co-cultures. Our results showed that increased CD40L levels led to a selective increased proliferation of IgM+ mBC, which did not class-switched, resulting in higher frequencies of IgM+ ASCs and a lower frequency of IgG+ ASCs. The IgG+/IgA+ mBCs were unaffected. We further compared the transcription of immune-related genes in IgM+ and IgG+ pre-plasmablasts cultured at high (500 ng/mL) and low (50 ng/mL) CD40L levels. In response to increased CD40L levels, both populations exhibited a core response to genes related to activation (TRAF1, AKT3, CD69, and CD80). However, they differed in genes related to cytokine/chemokine/homing interactions (CCL3/4/17, LTA, NKX2-3, BCL2 and IL21R) and cell-cell interactions (HLADR, CD40, and ICOSL), which were largely confined to IgG+ cells. Our findings revealed that in co-cultures with a high T-ratio, the response was similar to that found in cultures with high CD40L levels. These results suggest that IgG+ mBCs have a greater capacity for proliferation and T cell interaction, and weaker migration capabilities, leading to a preference for an IgG response over IgM in the short term. This adaptable response could fine-tune the memory repertoire with different functions of IgG versus IgM mBCs.

## 1 Introduction

Prior research describes memory B cells (mBCs) as circulating cells that patrol the body and that, after reactivation by the same pathogen, can re-enter the germinal center (GC) and provide a second burst of antibodies with higher affinity than a primary response. In recent years, fate decisions after reactivation have been debated, and recent publications have challenged certain dogmas noted above (1). Convincing evidence suggests that the population of mBCs is greater than that of naïve B cells in older individuals (2). This observation, combined with the notable long-term stability of mBCs (lasting decades) and their faster activation response *in vitro* compared to naïve cells, has led to the hypothesis that mBCs serve as more immediate precursors to plasma cells, which are responsible for both antibody-mediated defense and autoimmune disease (3). Upon encounter with cognate antigen, mBCs can be re-activated and re-enter a secondary GC or directly differentiate into antibody-secreting cells (ASCs) (3), which is relevant for most antigen-specific tissue-resident mBCs (4).

Reactivation of mBCs in secondary lymphoid organs likely occurs in recently described subcapsular proliferative foci, a special site within the lymph nodes enriched for mBCs, T follicular helper cells (TFH) providing CD40L and other signals, and antigen-presenting macrophages (5, 6). In humans, only a few of the hundreds of mBCs clones that are generated during the immune response are activated and develop into plasmablasts (PBs), and even fewer re-enter the germinal center (7). Interestingly, mBCs require additional signals such as Toll-like receptor and CD40 activation to achieve proper proliferative capabilities (8), which suggests that parameters other than affinity can modulate their response, that is, through interaction with T cells. However, the interaction between mBCs and T cells is not fully understood.

## 2 Materials and methods

### 2.1 Isolation of B and T cells

Peripheral blood samples (EDTA anticoagulated or serum tubes, BD Vacutainer System, BD Biosciences) were collected from healthy controls (n= 31, median=33, range=21-59). PBMCs were prepared by density gradient centrifugation using Ficoll-Paque PLUS (GE Healthcare Biosciences). B cells were enriched from PBMCs using the Pan B Cell Isolation Kit (Miltenyi), following the manufacturer’s instructions. Total T cells were enriched from PBMCs using the Pan T Cell Isolation Kit (Miltenyi), following the manufacturer’s instructions. Enriched B cells were stained and mBCs (CD19+CD20+CD27+, **Supplementary Figure S1A**) and naïve B cells (CD19+CD20+CD27-IgD+, **Supplementary Figure S1A**) were sorted using an ARIA II (BD Biosciences) instrument. mBCs purity (median 95.1%, range 84.1-98,7%), naïve purity (median 98.9%, range 75,6-100%).

### 2.2 B cell culture

Cells (at least 0.5x10^5 and no more than 1.5x10^5 cells) were cultured in 1 mL of culture medium in 48-well plates at 37°C and 5% CO2. The culture medium was prepared with IMDM culture medium (Thermo-Fisher), supplemented with 10% inactivated fetal bovine serum (Thermo-Fisher), 1% Penicillin/Streptomycin (Thermo-Fisher) and ITS liquid media supplement 100x (Sigma-Aldrich). Three sequential culture media were used, as previously described (8), to induce various stages of plasma cell differentiation, as previously described (1). From day 0 to day 4, to induce pre-PBs stage IMDM medium was supplemented with IL-2 (20 ng/mL, Miltenyi), IL-15 (20 ng/mL, Miltenyi), IL-10 (50 ng/mL, Miltenyi), CpG (2.5 µg/mL, ODN2006, Miltenyi), anti-BCR (0.5 µg/mL, Jackson Immunoresearch), and CD40L (0 – 5000 ng/mL, Miltenyi). From day 4 to 7, to induce PBs stage, IMDM medium was supplemented with IL-2 (20 ng/mL), IL-15 (20 ng/mL), IL-10 (50 ng/mL), and IL-6 (50 ng/mL, Miltenyi). Finally, from day 7 to 10, to induce early plasma cells, IMDM medium was supplemented with IL-15 (20 ng/mL), IL-6 (50 ng/mL), and IFN-α (10 ng/mL, Miltenyi). Cells were washed with pre-warmed PBS and counted after each change in the medium. Each step started with no more than 3×10^5 cells from the previous step. Supernatants from day 10 were stored and frozen at -20°C.

### 2.3 T - B co-culture

Naïve or mBCs B and total CD3+ T cells were enriched as indicated previously. Cells (1x10^5) were cultured separately in 96-well U-bottom plates in IMDM (V=250 µL) for 24 h at 37°C and 5% CO_2_. The cells were then washed and co-cultured in 250 µL of IMDM medium containing IL-2 (20 ng/mL), IL-15 (20 ng/mL), IL-10 (50 ng/mL), CpG (2.5 µg/mL), and anti-BCR (0.5 µg/mL) in 96-well U-bottom plates. Co-cultures were performed at T:B ratios of 1:1 (1x10^5 T cells/1x10^5 mBCs/naïve), 1:5 (0.2x10^5 T cells/1x10^5 mBCs/naïve), and 1:20 (0.04x10^5 T cells/1x10^5 mBCs/naïve) for 4 days at 37°C and 5% CO2. The supernatants were stored and frozen at -20°C.

### 2.4 Flow cytometry

To evaluate proliferation, mBCs and naïve B cells were previously stained with CFSE (Thermo-Fischer) or cell tracer violet (Thermo-Fischer), following manufacturer’s instructions. For staining, the cell suspensions were stained with blue live/dead dye (Thermo-Fischer) for 30 min at 4°C and then washed with MACS buffer (Miltenyi) and BSA (Miltenyi). The cells were fixed using BD Phosflow Lyse/Fix buffer (BD Biosciences) for 10 min at 37°C and permeabilized with BD Phosflow Perm Buffer II (BD Biosciences) for 60 min at 4°C. For staining, the cells were suspended in 40 μl of MACS buffer (Miltenyi) and 10 μl of Brilliant Buffer (BD Horizon). The cells were intracellularly stained for 60 min on ice and washed with MACS buffer and BSA. Flow cytometry analyses were performed using a BD FACS Fortessa (BD Biosciences). The cytometer setup, tracking beads (BD Biosciences), and rainbow calibration particles (BD Biosciences) were used to ensure comparable mean fluorescence intensities over time.

The following fluorochrome-labeled antibodies were used: BUV395 anti-CD14 (clone M5E2, 1:50, BD Biosciences), BUV395 anti-CD3 (clone UCHT1, 1:50, BD Biosciences), BV786 anti-CD27 (clone L128, 1:50, BD Biosciences), BV711 anti-CD19 (clone SJ25C1, 1:50, BD Biosciences), BV510 anti-CD20 (clone 2H7, 1:50, BD Biosciences), PE-CF594 anti-IgD (clone IA6-2, 1:500, Biolegend), APC–Cy7 anti-CD38 (clone HIT2, 1:1000, Biolegend), PE-Cy7 anti-IgG (clone G18-145, 1:500, BD Biosciences), anti-IgA–FITC (clone M24A, 1:250, Chemicon), BV421 anti-IgM (clone G20-127, 1:100, BD Biosciences), BUV737 anti-CD138 (clone MI15, 1:250, BD Biosciences), AF647 anti-TRAF1 (clone 1F3, 1:50, BD Biosciences), BV510 anti-CD3e (clone UCHT1, 1:50, BD Biosciences), PE/Dazzle 594 anti-CD137 (clone 4B4-1, 1:100, Biolegend), AF594 anti-TRAF6 (clone 326019, 1:50, R&D), and BV786 anti-CD40L (clone 24-31, 1:100, Biolegend). Antibody mixtures were prepared in Brilliant Stain Buffer (1:5, BD Biosciences). CountBrigh Absolute Counting Beads (Thermo-Fischer) were used to estimate number of cells.

### 2.5 Multiplex assay

The levels of immunoglobulins were measured in B cell supernatants (day 10) using the LEGENDplex Human Immunoglobulin Isotyping Panel (8-plex) on a V-bottom Plate (Miltenyi). Levels of soluble CD40L (sCD40L), BAFF, and APRIL were measured in T:B co-culture supernatants (day 5) using the LEGENDplex Human B Cell Activator Panel (3-plex) on a V-bottom Plate (Miltenyi). Immunoglobulin levels were normalized by cell number.

### 2.6 Sample preparation for gene expression profiling

mBCs were isolated and cultured for four days, as previously mentioned (B cell cultures), using two CD40L concentrations (50 and 500 ng/mL). The cells were then washed and stained (using DAPI, CD27, IgA, IgG, and IgM) to sort the CD27+IgG+IgM-IgA- and CD27+IgG-IgM+IgA-populations (**Supplementary Figure S1B**). Purity of IgG+ pre-PBs (median 95.05%, range 94.3 – 97.6%) and IgM+ pre-PBs (median 95.5%, range 93.8 – 99%). The cells were pelleted in a tube at 650 × g at 4°C and frozen at -80°C.

### 2.7 Library generation and sequencing

Samples were run on the HTG EdgeSeq Processor (HTG Molecular Diagnostics) using the HTG EdgeSeq Immune Response Panel (HTG Molecular Diagnostics) following the manufacturer’s instructions. Briefly, cells were lysed using proteinase K and denatured oil with an excess of nuclease-protection probes complementary to their targets. S1 nuclease, then removed un-hybridized probes, and RNAs leaving behind nuclease protection probes hybridized to their targets in a 1-to-1 ratio. Samples were individually barcoded through PCR to add adapters and molecular barcodes, individually purified using AMPure XP beads (Beckman Coulter), and quantified using a KAPA Library Quantification Kit (KAPA Biosystems). Libraries were sequenced on an Ion Torrent platform (Thermo-Fisher). Quality control, standardization, and normalization were performed by HTG and were provided to the investigators. Quality control criteria as determined by the manufacturer were met for all samples.

### 2.8 Gene expression profiling

Gene expression analysis was performed using R (version 4.2.2, R Foundation for Statistical Computing) (9) and DESEQ2 package (10). The model design used for this analysis contained three variables: cell type (IgM/IgG), CD40L concentration (low/high), and their interactions (cell type: CD40L concentration). IgM+ cells and low CD40L level were chosen as reference groups for the analysis.

Genes were considered differentially expressed if they had a p value < 0.1 and an absolute fold change > 1.5. Five lists of differentially expressed genes (DEG) were generated: 1) DEG in CD40L low levels (**Supplementary Table 1**), 2) DEG in CD40L high levels (**Supplementary Table 2**), 3) DEG in IgM+ cells (**Supplementary Table 3**), 4) DEG in IgG+ cells (**Supplementary Table 4**), and 5) DEG with interaction of factors (**Supplementary Table 5**).

Enrichment analysis was performed in R with the clusterProfiler package (11) using terms from the KEGG ontology and pathway (12). Visualization of data was performed using cnetplot and dotplot from the clusterProfiler package, as well as the ggplot2 package (13). Cnetplot was constructed combining data from **Supplementary Tables 3** and **4**. Dotplot was constructed with DEG listed in **Supplementary Tables 1** and **2** for **Figure 3C**, and **Supplementary Table 5** for **Figure 4F**.

### 2.9 Statistical analysis

Statistical analyses were performed using GraphPad Prism (v9.5, GraphPad Software). To test the data among the multiple CD40L concentrations, we performed non-parametric Kruskall Wallis tests, with Dunn´s post-test. To compare paired data, we performed paired non-parametric Wilcoxon test. To compare the results from co-cultures, we used non-parametric pairwise Friedman tests, with Dunn´s post-test. To compare grouped data, such as CD40L concentration (low/high) and cell type (IgM/IgG/IgA), T:B ratios (1:20/1:5,1:1) and cell types (IgM/IgG/IgA); we performed Two-way ANOVA tests, with Tukey´s post-test. Differences with p-values <0.05 were considered statistically significant (* p<0.05, ** p<0.01, *** p<0.001, **** p<0.0001).

## 3 Results

### 3.1 Increased CD40L concentration leads to reduced IgG+ and increased IgM+ plasmablasts

**Figure 1:**
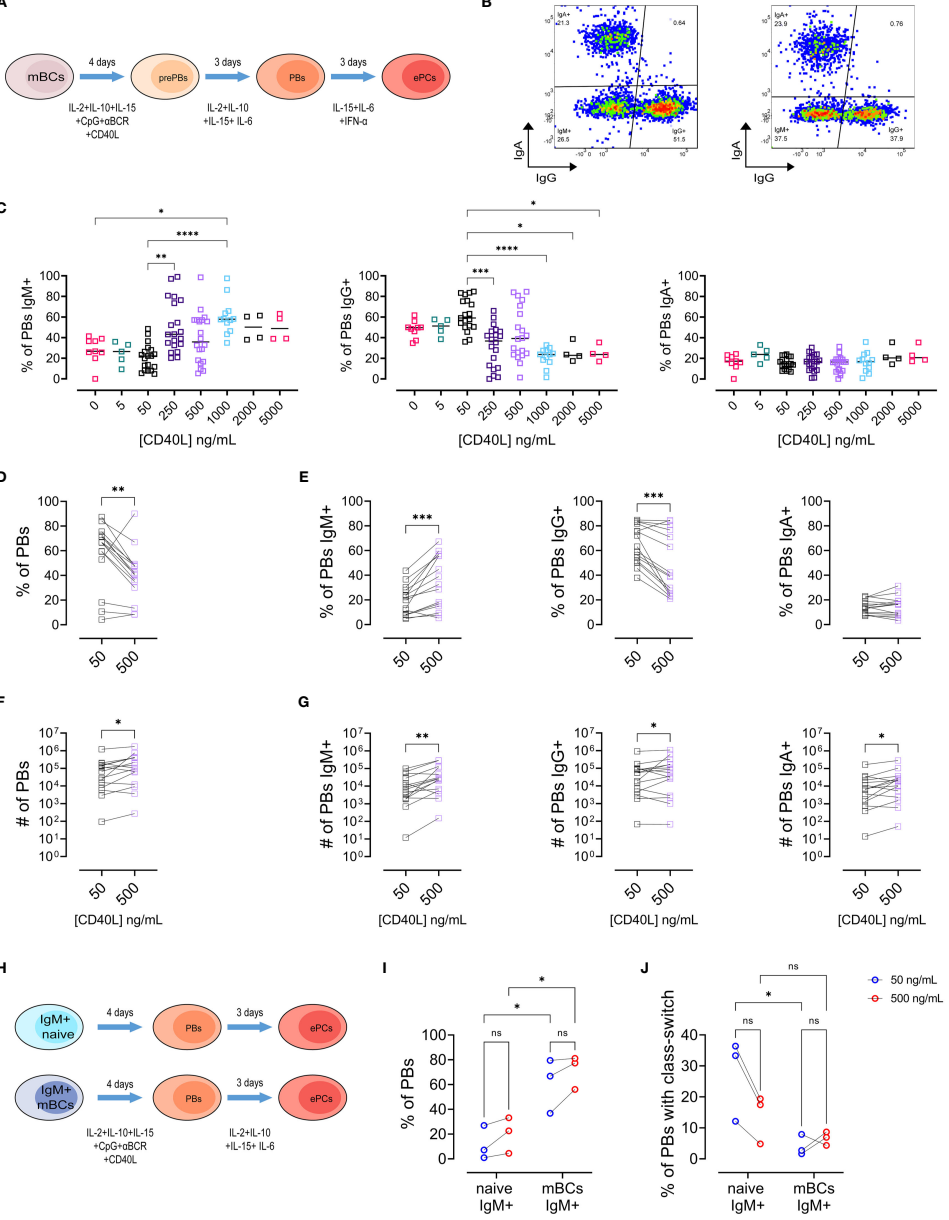
Increasing levels of CD40L have divergent effects on the frequencies of IgG+ and IgM+ plasmablasts. **(A)** Representative scheme of different steps of culture of memory B cells (mBCs) to differentiate into pre-plasmablasts (prePBs) at day 4, plasmablasts (PBs) at day 7, and early plasma cells (early PCs) at day 10. **(B)** Representative pseudocolor plot of PBs populations based on IgG and IgA expression in cultures with low (50 ng/mL, left) or high (500 ng/mL, right) CD40L. **(C)** Frequency of Plasmablast with IgM+, IgG+, or IgA+ phenotype among different CD40L levels. (**D, E)** Frequency of **(D)** Plasmablast and **(E)** Plasmablast with IgM+, IgG+, or IgA+ phenotype in cultures with 50 and 500 ng/mL of CD40L. **(F, G)** Estimated number of **(F)** Plasmablast and **(G)** Plasmablast with IgM+, IgG+, or IgA+ phenotype in cultures with 50 and 500 ng/mL of CD40L. **(H)** Representative scheme of different steps of culture of sorted IgM+ naïve and IgM+ mBCs to differentiate into PBs at day 7. **(I)** Frequency of PBs in sorted population cultures on day 7. **(J)** Frequency of PBs with class-switch. **(C)** Non-parametric Kruskal–Wallis test with Dunn’s post-test. **(D–G)** Paired non-parametric Wilcoxon test. **(I, J)** Two-way ANOVA with Tukey’s *post-hoc* test. *p<0.05, **p<0.01, ***p<0.001, ****p<0.0001. ns, Not significant.

We performed a series of experiments based on a previously published protocol for differentiation of mBCs into pre-PBs (day 4), plasmablasts (PBs, day 7), and early plasma cells (early PCs, day 10) (**Figures 1A, B**) (9). We used CD40L as a model of T cell interaction with mBCs during PBs/early PCs differentiation and evaluated the isotype preference of sorted mBCs and naïve B cells for a series of CD40L concentrations (0 – 5000 ng/mL, **Figures 1C–F**). At the PBs stage, no differences were observed in viability, frequency of PBs, and number (**Supplementary Figures S2A–C**) between the CD40L levels evaluated. Most notably, the analysis of IgM, IgG, and IgA isotypes by PBs showed a general increase in the frequency of IgM+ PBs alongside with a reduction in the frequency of PBs with an IgG+ phenotype in cultures with higher CD40L concentrations (**Figure 1C**). Therefore, we chose 50 ng/mL (low) and 500 ng/mL (high) of CD40L for further detailed analysis.

We observed decreased frequency of PBs with high CD40L (**Figure 1D**). We found, similar to observed before, an increased frequency of IgM+ and a decreased of IgG+ populations with the increase in CD40L (**Figure 1E**). No differences were observed for the IgA+ PBs (**Figure 1E**). As expected, we found an increase in the number of PBs (**Figure 1F**), as well as in the three PBs populations (**Figure 1G**) with high CD40L levels. These observations for IgG+ and IgM+ PBs were also observed in cultures with a different recombinant CD40L without a cross-linking antibody (**Supplementary Figures S2E, F**). Notably, these effects were only observed in mBCs cultures, but not in naïve B cell cultures (**Supplementary Figure S3**).

Next, we evaluated at early PC stage on day 10 of mBCs culture (**Supplementary Figure S4A**). To corroborate early PC phenotype in addition to CD27, CD28, and CD138 expression, in some cultures we evaluated EZH2. We observed lower EZH2 expression in early PCs compared to PBs (**Supplementary Figure S4B**), regardless of the concentration of CD40L used. Then, early PCs were classified into 3 subsets according to IgA, IgG, and IgM phenotype (**Supplementary Figure S4C**). No differences were observed in viability (**Supplementary Figure S4D**) or in the frequency and number of early PCs (**Supplementary Figure S4E**) with high CD40L concentration. We observed a similar reduction in frequency of the IgG+ cells, as well as an increase in the IgM+ population as observed on day 7 in PBs (**Supplementary Figure S4F**). Also, as expected, we observed an increased number of early PCs, as well in all the early PCs with the evaluated isotypes (**Supplementary Figures S4G, H**). These results suggest that mBCs can react to a range of CD40L amounts, leading to different outcomes in terms of isotype preference at the PBs and early PCs stages.

Subsequently, we evaluated the production of different immunoglobulins (IgA, IgG1, IgG2, IgG3, IgG4, IgD, IgE, and IgM) by early PCs. No differences in the immunoglobulin production per cell between the different concentrations of CD40L were observed (**Supplementary Figure S5**), suggesting that the initial stimulation with CD40L impacts on cellular expansion with isotype preference but not on immunoglobulin production/secretion per cell.

### 3.2 Low induction of class-switching in IgM+ mBCs compared with naive B cells

The changes in the frequencies of IgM+ and IgG+ cells observed in the cultures in response to CD40L could be due to differences in the susceptibility to proliferation or class-switch induction. Therefore, we sorted IgM+ naïve and IgM+ mBCs and cultured them with low or high CD40L levels until the pre-PB, PB, and early PC stages (**Figure 1H** and **Supplementary Figures S6A–C**). As expected, we observed higher frequency of PBs (**Figure 1I**) but reduced class-switch (**Figure 1J**) in IgM+ mBCs compared to naïve cultures. Interestingly, IgM+ mBCs cultures showed no differences in class switching between low and high CD40L levels (**Figure 1J**). No differences were observed at stages pre-PBs or early PCs (**Supplementary Figure S6**). These data indicate that the observed differences in mBCs cultures cannot be explained by class-switch induction upon CD40 activation only.

### 3.3 Higher levels of CD40L trigger initial cell division of IgM+ mBCs but reduces capacity to undergo multiple rounds

**Figure 2:**
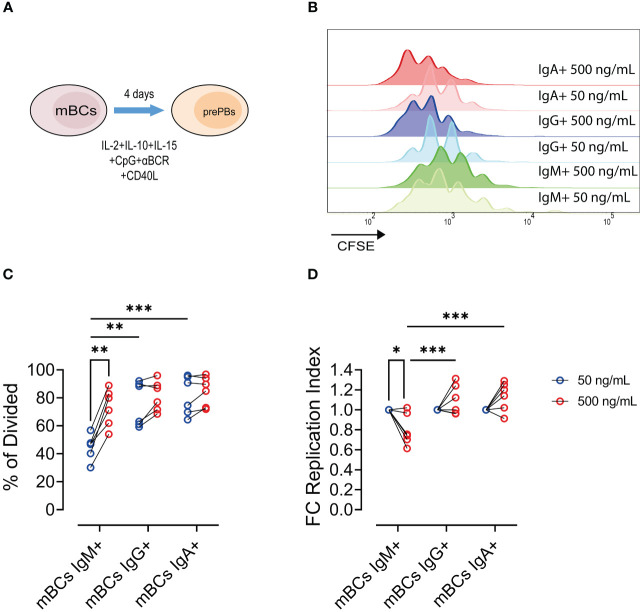
Increase of CD40L levels leads to increase frequency of proliferative IgM+ mBCs but not in IgA+/IgG+ memory B cells. **(A)** Representative scheme of culture of mBCs or naïve B cells with 50 and 500 ng/mL CD40L for 4 days. **(B)** Representative histogram of CFSE expression among IgM+, IgG+, and IgA+ cells from mBCs cultures on day 4. **(B)** Frequency of IgM+, IgG+, and IgA+ cells in mBCs cultures on day 4. **(C)** Frequency of divided cells among IgM+, IgG+, and IgA+ from mBCs from cultures on day 4. **(D)** Fold change of replication index among IgM+, IgG+, and IgA+ mBCs cells from cultures on day 4. Two-way ANOVA test with Tukey´s post-test. Data from six donors from two independent experiments. *p<0.05, **p<0.01, ***p<0.001.

To evaluate the proliferative capabilities, we evaluated the response upon CD40 stimulation at the pre-PBs stage through proliferative modelling (**Figure 2A**). We cultured mBCs or naïve B cells with low and high CD40L levels until the pre-PBs stage (**Figure 2B**).

In mBCs cultures, for high CD40L levels we found an increase in the frequency of divided IgM+ pre-PBs compared to IgG+ or IgA+ cells (**Figure 2C**). The frequency of dividing cells with low CD40L levels in IgM+ mBCs was lower than observed in IgA+ and IgG+ cells, but at high CD40L levels in cultures the frequencies of divided cells were similar in any of the populations (**Figure 2C**). Interestingly, we found a reduction in the expansion of the responding cells (cells which went into proliferation), also known as replication index, in IgM+ mBCs cultured with high CD40L levels compared with low CD40L levels (**Figure 2D**). However, in IgG+ and IgA+ cells, we observed a tendency to increase in this fold expansion (**Figure 2D**). We found a higher replication index in IgG+ and IgA+ pre-PBs compared to IgM+ population in cultures with high CD40L levels (**Figure 2D**).

These results suggest that, in response to higher CD40L levels, more IgM+ mBCs cells are being triggered to divide but their capacity to undergo multiple rounds of division is limited, resulting in a lower average number of divisions per cell across the population. These changes were not found for IgG+ and IgA+ mBCs populations.

### 3.4 At transcriptional level, IgM+ vs IgG+ mBCs showed differential response for equal CD40L stimulation

**Figure 3:**
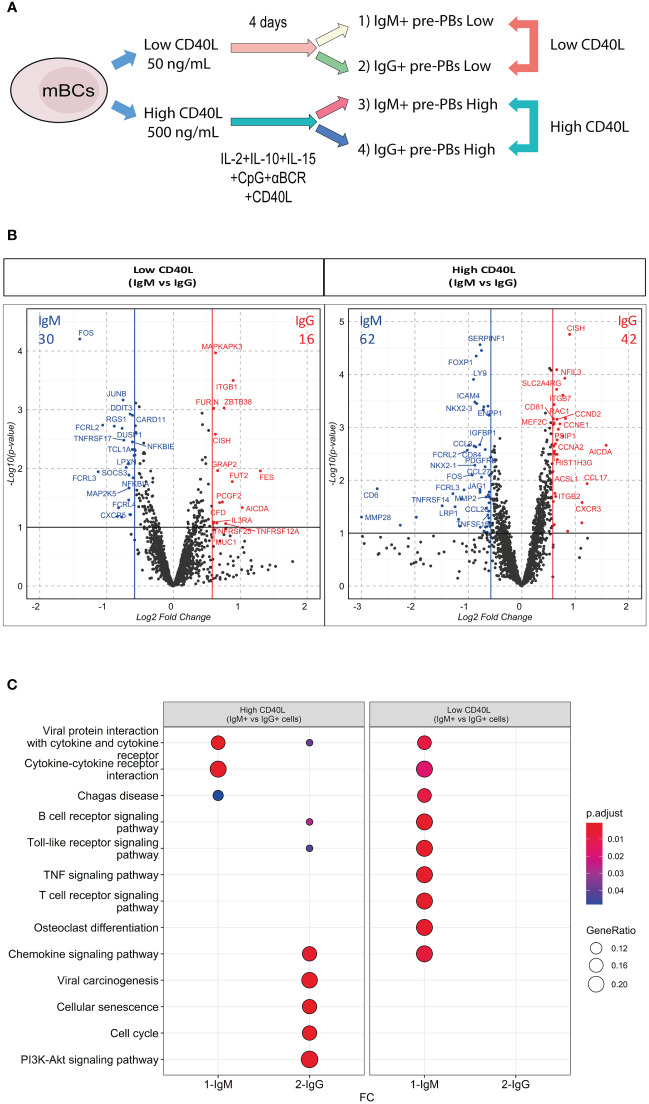
IgM+ and IgG+ mBCs showed differential response to equal CD40L levels. **(A)** Representative scheme of culture of memory B cells (mBCs) to carry immune targeted RNAseq analysis. After 4 days of co-culture with low or high CD40L levels, CD27+IgM+ and CD27+IgG+ pre-PBs were sorted. Comparison of IgM vs IgG pre-PBs in Low CD40L and High CD40L levels were analyzed. **(B)** Volcano plots showing differentially expressed genes (DEG) in Low CD40L (left) or High CD40L (right) conditions. Ref=IgM. **(C)** KEGG pathway enrichment of DEG from Down- and Up-regulated genes in Low CD40L and High CD40L comparisons. Lists of genes are presented in **Supplementary Tables 1** and **2**.

To understand the effect of CD40L on mBCs differentiation, we analyzed the effects of low or high CD40L on IgM+ and IgG+ pre-PBs at the transcriptomic level. For this, mBCs were cultured as indicated for 4 days, and then CD27+IgM+ and CD27+IgG+ pre-PBs were sorted (**Figure 3A**, **Supplementary Figure S6A**). Transcriptomic analysis was performed in these four populations from six donors, using the HTG EdgeSeq Immune Response Panel with 2002 immune response mRNA targets (**Figure 1B**, **Supplementary Figure S6B**).

We initially compared IgM+ vs IgG+ pre-PBs from cultures with low CD40L levels and analogously compared for the high CD40L levels condition (**Figure 3A**). In low CD40L levels, we found 30 up-regulated genes in IgM+ pre-PBs compared with IgG+ cells, alongside with 16 up-regulated genes in IgG+ pre-PBs compared with IgM+ pre-PBs (**Figure 3B** left). In high CD40L conditions, we identified 62 up-regulated genes in IgM+ cells and 42 in IgG+ pre-PBs (**Figure 3B** right). The higher amount of DEGs found with higher levels of CD40L suggest distinct transcriptional responses dependent on increased T cell involvement.

Bringing together the IgM+ vs IgG+ DEG analysis in both CD40 high and CD40 low conditions, we found low shared KEGG enrichment (**Figure 3C**). In low CD40L levels, DEG in IgM+ cells showed KEGG enrichment mainly in pathways related to activation (such as B cell receptor, TLR, TNF) (**Figure 3C**). In high CD40L levels, IgM+ cells showed enrichment for cytokine interactions. In contrast, IgG+ cells showed no enrichment in low CD40L levels, and enrichment in high CD40L conditions for cellular senescence, cell cycle and PIK3-Akt pathways (**Figure 3C**). These results suggest IgM+ and IgG+ pre-PBs respond differently to equal CD40L levels, possibly due to intrinsic differences of the initial mBCs.

### 3.5 CD40L concentration alters mBCs populations at the transcription level

**Figure 4:**
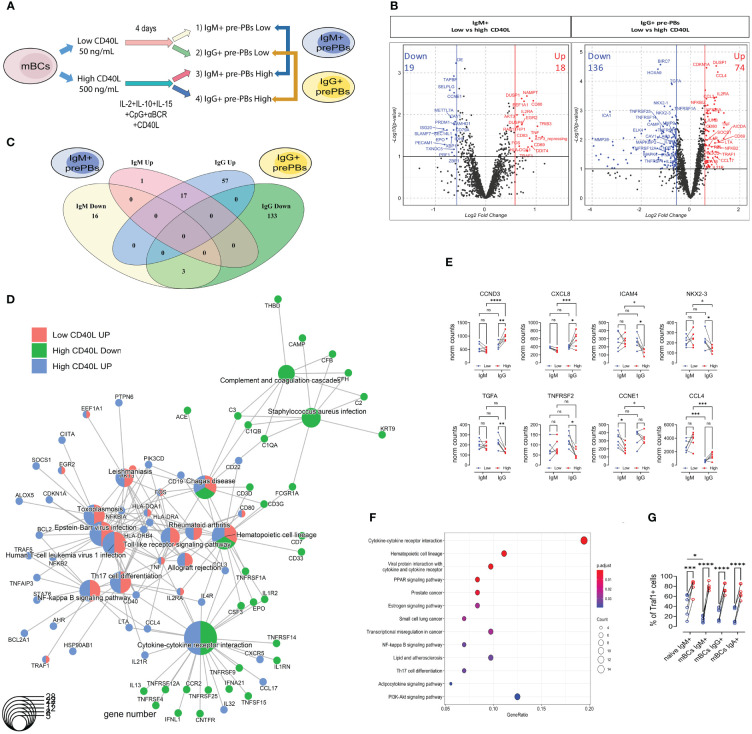
IgM+ and IgG+ memory B cells share a common basic core response to high CD40L levels but differ in genes related to cytokines/chemokines, mainly in IgG+ mBCs. **(A)** Representative scheme of culture of memory B cells (mBCs) to carry immune targeted RNAseq analysis. After 4 days of co-culture with low or high CD40L levels, CD27+IgM+ and CD27+IgG+ pre-PBs were sorted. **(B)** Volcano plots showing differentially expressed genes (DEG) in IgM+ (left) or IgG+ (right) comparisons. Ref=Low CD40L. **(C)** Venn Diagram analysis of 37 differentially expressed genes (DEG) from IgM, and 210 DEG from IgG comparisons. The list of genes is presented in **Supplementary Tables 3** and **4**. **(D)** Cnetplot of DEG, with genes and functional categories (KEGG enrichment) encoded as pies to distinguish different gene clusters. DEG comes from IgM (low vs high) and IgG (low vs high) comparisons. **(E)** Data for selected genes with interaction of factors (cell type and CD40L concentration). A list of the genes is presented in **Supplementary Table 5**. **(F)** KEGG pathway enrichment analysis of DEG with interaction of factors. **(G)** Frequency of Traf1+ cells in IgM+, IgG+, and IgA+ cells from mBCs cultures with 50 and 500 ng/mL of CD40L. Two-way ANOVA with Tukey’s *post-hoc* test. *p<0.05, **p<0.01, ***p<0.001, ****p<0.0001. ns, Not significant.

As a next step, we compared the response to the different culture conditions (low CD40L vs high CD40L) in both populations of pre-PBs (IgM+ and IgG+) (**Figures 4A, B**). In IgM+ mBCs, we observed 37 DEG, of which 18 were upregulated and 19 were downregulated at high CD40L levels compared to low CD40L levels (**Figures 4B, C**, and **Supplementary Table 3**). For IgG+ mBCs, we identified 210 DEG, of which 74 were upregulated and 136 were downregulated (**Figures 4B, C** and **Supplementary Table 4**).

Of the 75 upregulated genes related to high CD40L levels, only one (AKT3) was exclusively found in IgM+ mBCs, whereas 17 (such as CD69, CD80, TNF, and TRAF1) were shared between IgM+ and IgG+ mBCs (**Figure 4C**, **Supplementary Tables 3** and **4**). The other 57 upregulated genes were found only in IgG+ mBCs (such as CD40, CXCR5, HLA-DR, NFKB2, PAX5, and TRAF5) (**Figure 4C** and **Supplementary Table 4**).

Of the 152 downregulated genes, three (CAV1, EPO, and PECAM1) were shared between IgM+ and IgG+ mBCs, whereas 16 (such as CD79A and SAMHD1) occurred only in IgM+ mBCs, and 133 (such as CCR2, GAB2, NKX2-3, TNFRSF25, TNFRSF4, and TNFRSF9) exclusively in IgG+ mBCs (**Figure 4C**, and **Supplementary Tables 3** and **4**).

With the up- and down-regulated DEG from both IgM+ or IgG+ pre-PBs in response to high CD40L levels (four groups), we performed a combined KEGG enrichment analysis and clustered genes by KEGG enrichment (cnetplot, **Figure 4D**). There was no KEGG enrichment of downregulated genes in IgM+ pre-PBs (**Figure 4D**). Contrary to what we observed for each CD40L condition (IgM+ vs IgG+, **Figure 4**), three clear clusters were identified (**Figure 4D**). The first is structured around cytokine-cytokine receptor interactions, with genes down- (such as CCR2, TNFRSF14, and TNFRSF25) and upregulated (such as CXCR5, CCL17, and CCL4) only in the IgG+ pre-PBs. The second cluster comprised only downregulated genes (such as related to complement) from the IgG+ pre-PBs (**Figure 4D**). The third cluster contained upregulated genes from both IgM+ and IgG+ pre-PBs. This cluster contained genes related to the NF-κB, TLR, and TNF pathways as well as T cell interactions (**Figure 4D**). In this cluster, we identified genes such as TRAF1, TRAF5, CD40, HLA-DR, NFKB2, and BCL2 (**Figure 4D**).

These findings underscore the complexity of B cell differentiation, revealing that IgG+ mBCs not only share common activation pathways with IgM+ cells but also engage in unique transcriptional programs, particularly in response to cytokine signaling, which may reflect their specialized roles in the immune response.

### 3.6 Interaction of CD40L levels and cell type factors reveals enrichment of cytokine/chemokine interactions

To understand the responses at the transcriptomic level, we performed an interaction analysis of the two factors: cell type (factor 1: IgM+/IgG+) and CD40L stimulation (factor 2: low/high) (**Figure 4E**). We found 96 genes that interacted with one or more factors (**Supplementary Figure S7C** and **Supplementary Table 5**).

Some of these DEG showed increased expression in IgG+ but not in IgM+ cells, such as CCND3 (also known as cyclin-D3) and CXCL8 (**Figure 4E**). Others showed decreased expression in IgG+ but no changes in IgM+, such as ICAM4, NKX2-3, and TNFRSF2 (**Figure 4F**). CCL3 and CCL4 showed higher expression levels in IgM+ pre-PBs but did not show differences in response to high CD40L levels (**Figure 4E**). These genes with interaction of factors showed KEGG enrichment in cytokine-cytokine receptor interaction and cell activation pathways (NF-κB, PPAR, and PI3K-Akt) (**Figure 4F**).

The results suggest DEG which had an effect of one factor (CD40L levels) according on the conditions in the other factor (mBC type), showed an enrichment of cytokine/chemokine interactions.

### 3.7 Increased frequency of Traf1+ B cells in cultures with high CD40L levels

Activation of the CD40 pathway can lead to the activation of both canonical and non-canonical NF-κB pathways. The canonical pathway, which is rapid and transient (14), is employing TRAF6. Interestingly, there was no difference in the frequency of TRAF6+ cells between CD40L concentrations (**Supplementary Figure S7D**). The non-canonical pathway, described as slow and persistent (14), is regulated by TRAF1. We observed an increase in the frequency of Traf1+ cells in cultures with higher CD40L concentrations among the three populations (IgM+, IgG+, and IgA+) in the mBCs cultures (**Figure 4G**). These results suggest that both mBCs and naïve B cells cultured with high CD40L levels activate CD40 through the non-canonical pathway as reflected by the upregulation of Traf1.

### 3.8 Similar behavior of Ig preference observed in co-cultures of T and mBCs

**Figure 5:**
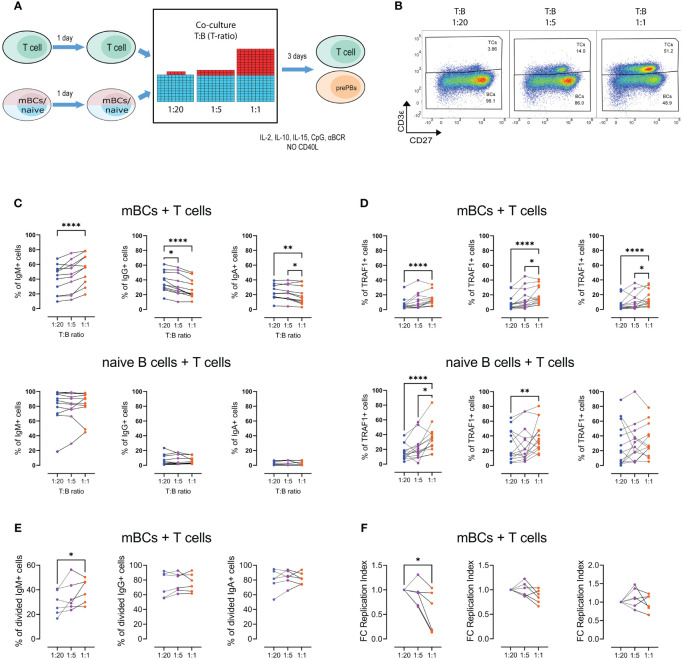
Increasing the T-cell ratio results in divergent effects on the frequency of IgG+ and IgM+ pre-plasmablasts. **(A)** Representative scheme of co-culture of memory B cells (mBCs) or naïve B cells with T cells, at T:B ratios of 1:1, 1:5, and 1:20 (B cell numbers were always fixed). After four days of co-culture, the cells were analyzed by flow cytometry. **(B)** Representative pseudocolor plots of the different T:B cell ratios in co-cultures. **(C)** Frequency of IgM+, IgG+, and IgA+ cells at the end of mBCs (top) or naïve B cells (bottom) co-cultured with T cells at the indicated T:B ratios. **(D)** Frequency of Traf1+ cells in IgM+, IgG+, and IgA+ B cells from co-culture of T cells with mBCs (top) or naïve B cells (bottom). **(E, F)** Frequency of **(E)** divided cells and **(F)** fold change of replication index (right) among IgM+, IgG+, and IgA+ cells from mBCs co-cultures among indicated T:B ratios. Friedman test with Dunn’s post-test. *p<0.05, **p<0.01, ****p<0.0001.

Given the results for CD40L concentrations, we evaluated the direct role of T cell on cultured mBCs. To do this, we co-cultured autologous T cells with mBCs or naïve B cells using a stimulation similar to that of enriched mBCs cultures, but in the absence of CD40L (**Figure 5A**). Co-cultures were performed using fixed numbers of T cells at T:B ratios of 1:20, 1:5, and 1:1 (**Figure 5A**). On day 4 of culture (**Figure 5B**), the expressions of IgM, IgG, and IgA were evaluated (**Figure 5C**, **Supplementary Figures S8A, B**).

We found that a higher number of B cells in the culture, corresponding to decreased amounts of CD40L, resulted in an increase in the frequency of IgM+ cells (**Figure 5C**), like those observed in the enriched cultures. Similarly, we observed the opposite effect in the frequency of cells with the IgG+ phenotype (**Figure 5C**). Interestingly, we also found a decrease in the IgA phenotype (**Figure 5C**), which was not observed in the mBCs cultures. In addition, these effects were not observed in co-cultures with naïve B cells (**Figure 5C**). We also observed increased frequency of Traf1+ B cells with higher T-ratios, both in co-cultures with mBCs and naïve B cells (**Figure 5D**).

At the end of the co-culture, the expression of the activation markers CD40L and CD137 was evaluated on T cells. No differences were found in the frequency of CD40L+ and CD137+ T cells in mBCs and naïve cultures (**Supplementary Figure S8C**). There was no difference in the levels of soluble CD40L in the supernatants of the co-cultures, either with mBCs or naïve cells (**Supplementary Figure S8D**). In addition, no differences were observed in BAFF or APRIL in the supernatants of co-cultures with mBCs (**Supplementary Figure S8D**).

In IgM+ cells, we observed an increased frequency of proliferating cells in response to increase in T-ratio present (**Figure 5E**). No changes were observed for IgG+ and IgA+ cells (**Figure 5E**). We also observed a decreased replication index with an increase in the T-ratio, an effect that was not observed for IgG+ and IgA+ populations (**Figure 5F**). IgG+ and IgA+ cells showed higher values than IgM+ cells in terms of the frequency of divided cells and the replication index (**Figure 5F**).

These results suggest that similar to observed with increased CD40L levels, the fraction of IgM+ mBCs that enter in proliferation to higher T-ratio also increase but had a lower proliferation potential in a T cell dose dependent manner.

## 4 Discussion

**Figure 6:**
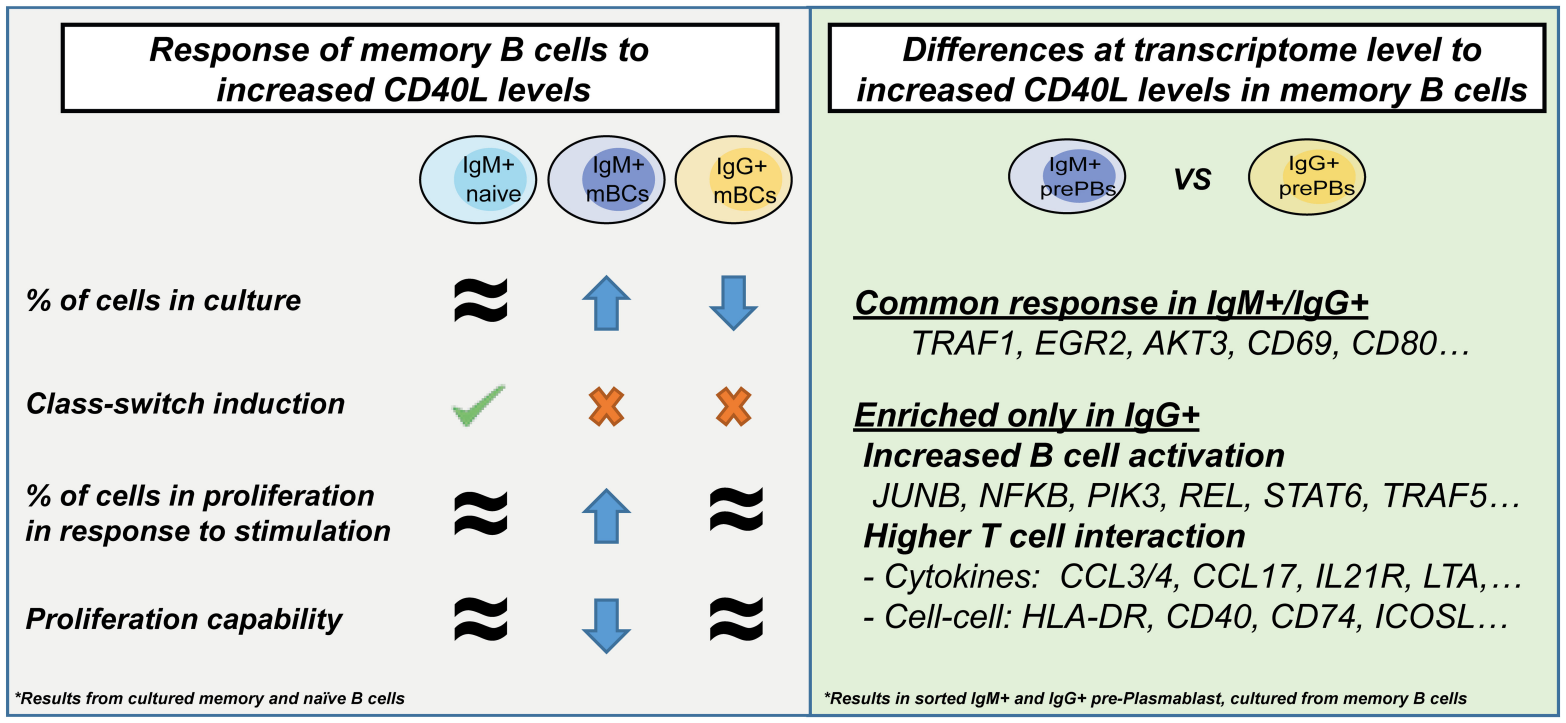
Graphical summary of the results.

In the current study, we found that the amplitude of CD40L available tuned mBC fate, increased expression of Traf1, and modulated the proliferation of mBC populations. In addition, IgM+ and IgG+ populations, in response to high CD40L levels, showed a similar core response (characterized by activation-related genes) but also differed in genes related to cytokine/chemokine interactions (**Figure 6**).

As previously mentioned, mBCs co-localize with antigen-presenting macrophages and TFH cells (6), which express higher levels of CD40L than naïve or effector CD4+ T cells (15). However, the expression of CD40L is not uniform. Some TFH cells exhibit CD40L expression levels like naïve/effector CD4+ T cells while other populations carry a 2-3 log higher expression (15), suggesting the presence of “low” and “high” CD40L stimulation *in vivo* that co-localized *in situ* with mBCs.

B cells cultured with cell lines expressing high CD40L levels showed an increased frequency of mBCs with high CD80 expression compared to cells cultured with low/medium CD40L expression (15). The authors also showed that B cells with high CD80 expression had a BCR with a higher affinity and were more prone to differentiate into PCs than GC B cells (15). We observed upregulated transcriptional levels of CD80 in both IgM+ and IgG+ mBCs cultured with high CD40L levels compared to those exposed to low CD40L levels, suggesting a similar effect of CD40 signaling on human pre-PBs.

The response to CD40L could also reflect early and late response scenarios. In the initial response, there is a high expression of antigen by follicular dendritic cells (FDCs), combined with relatively low competence for T cell interaction and survival signals (16). However, this immune response must culminate at some point (17). FDCs can retain antigens for at least 56 days after immunization in a GC-dependent or GC-independent manner, but at a lower expression than that observed at an early stage (18). At a later stage, B cells compete for antigen and T cell interactions. If there is a reduction in CD40L levels, our data suggest a progressive increase in the frequency of the IgM+ population (together with the concomitant reduction in IgG/IgA) due to the increase in proliferative capability (as observed in co-cultures).

Unlike the canonical NF-κB pathway, the non-canonical NF-κB pathway shows robust activation upon high CD40L stimulation (19). We found an increased frequency of Traf1+ cells in cultures with higher CD40L concentrations as well as in co-cultures with higher T-ratios, suggesting a role for the non-canonical response. However, the change in the frequency of IgA+ pre-PBs in the co-cultures, but not in the enriched B cell cultures, as well the change in frequency of IgM+ cells which undergo proliferation with higher CD40L levels but not with higher T-ratio, suggests that the interaction between T cells and mBCs is more complex than CD40L effects alone. The non-canonical response can also be induced by other signals such as RANKL and LTβR (20). However, the involvement of other pathways needs to be delineated.

Among the downregulated genes in IgG+ mBCs, we found NKX2-3 and MST1. NKX2-3 regulates B cell dynamics (21), enhancing the migration, polarization, and homing of B cells to splenic and extra-nodal tissues (21). Mst1 Kinase is required for follicular B cell homing through the positive regulation of BCR signaling (22). CCL3 and CCL4, which were upregulated in IgG+ mBCs, act as chemoattractants for T cells (23). CCL17 was also increased in IgG+ mBCs, and its receptor (CCR4) can be found in TFH (24). We also observed the upregulation of BCL2 in IgG+ cells. Mice overexpressing Bcl2 in B cells showed retention of ASCs in foci after NP-KLH immunization (25). Plasma cells generated from mBCs in the subcapsular foci decrease in frequency one week after recall, suggesting that this response generates mainly short-lived plasma cells (6). These observations suggest that IgG+ mBCs proliferate and differentiate relatively fast into plasma cells *in situ*.

We also found upregulation of AICDA only in IgG+ cells. Aicda is the key component responsible for modifying the specificity of B cells and for class switching. We found low induction of class-switching, suggesting that the mBCs isotype repertoire is largely predefined by the primary naive B cell instruction. In the recall response, mBCs can potentially undergo extrafollicular activation and therefore avoid additional GC rounds as shown for flavivirus-specific mBCs (26). However, after secondary immunization, both tetanus-specific mBCs and ASCs have unique mutations, indicating that committed precursors still retain the capacity for somatic hypermutations (27). These data suggest that some IgG+ mBCs interacting with high-CD40L-expressing TFH cells could re-enter in a GC-like reaction under certain conditions that need to be determined.

Some questions regarding the interaction between mBCs and T cells remain unanswered. What additional mechanisms beyond CD40 activation mediate this interaction? There are additional factors that could affect this process such as IL-21, which is produced by TFH cells. How does T cell activation affect the mBCs differentiation? Can mBCs undergo a rechallenge into long-lived ASCs repertoires? A limitation of the present study is the limited availability of other mBCs populations, such as double-negative (DN) B cells and CD11c+ B cells, which prevented us from evaluating them. We cannot rule out results might be different for these atypical memory populations.

The overall different response to CD40 stimulation by mBCs versus naive B cells indicates that mBCs are preferentially expanded selectively based on their Ig isotype expression with limited switching in contrast to naive B cell responses. Our results support the hypothesis that IgG+ mBCs, in response to high CD40L levels (or T-ratio), could decrease their migration capabilities, improve their ability to attract T cells, and differentiate and proliferate at the place of activation. IgM+ pre-PBs, responding to intense T cell interaction, would also proliferate *in situ* but show a reduced proliferative response and more prone to migration than IgG pre-PBs.

This effect would favor amplification in the short term of an IgG response instead of IgM *in situ*. Interestingly, mice re-challenged with influenza vaccine showed resident mBCs migration, rapid differentiation to ASCs and their proliferation (visible at day 4), and an increased frequency of IgG+ ASCs population (while IgA and IgM remained stable) at infected foci (28). These different cellular responses support the adaptation of a primary and secondary immune response while generating sufficient stability together with mechanisms to avoid depletion of cellular memory reservoirs by an ongoing immune response.

## Data availability statement

The original contributions presented in the study are publicly available. This data can be found here: https://figshare.com/s/d4333df6ca7f3c989bc2.

## Ethics statement

The studies involving humans were approved by Charité’s Ethics Committee, Charité Universitätsmedizin Berlin. The studies were conducted in accordance with the local legislation and institutional requirements. The human samples used in this study were acquired from primarily isolated as part of your previous study for which ethical approval was obtained. Written informed consent for participation was not required from the participants or the participants’ legal guardians/next of kin in accordance with the national legislation and institutional requirements. Written informed consent was obtained from the individual(s) for the publication of any potentially identifiable images or data included in this article.

## Author contributions

HR: Formal analysis, Methodology, Writing – original draft, Writing – review & editing. AS: Methodology, Writing – review & editing. TL: Formal analysis, Methodology, Writing – review & editing. MC: Data curation, Methodology, Writing – review & editing. AW: Methodology, Project administration, Writing – review & editing. FS: Formal analysis, Methodology, Writing – review & editing. JR: Methodology, Writing – review & editing. VD: Formal Analysis, Writing – review & editing. AL: Conceptualization, Formal analysis, Validation, Writing – review & editing. TD: Conceptualization, Funding acquisition, Resources, Supervision, Writing – review & editing. ES: Conceptualization, Data curation, Formal analysis, Funding acquisition, Methodology, Project administration, Resources, Supervision, Validation, Writing – review & editing.
