# Supplementary material for: Differential response of IgM and IgG memory B cell populations to CD40L: insights of T cell – memory B cell interactions
Source: Front Immunol. 2024 Jul 10;15:1432045. doi: 10.3389/fimmu.2024.1432045 (PMC11266000; doi:10.3389/fimmu.2024.1432045)
Supplement: Supplementary file 1 [file Presentation_1.pptx]

## Slide 1
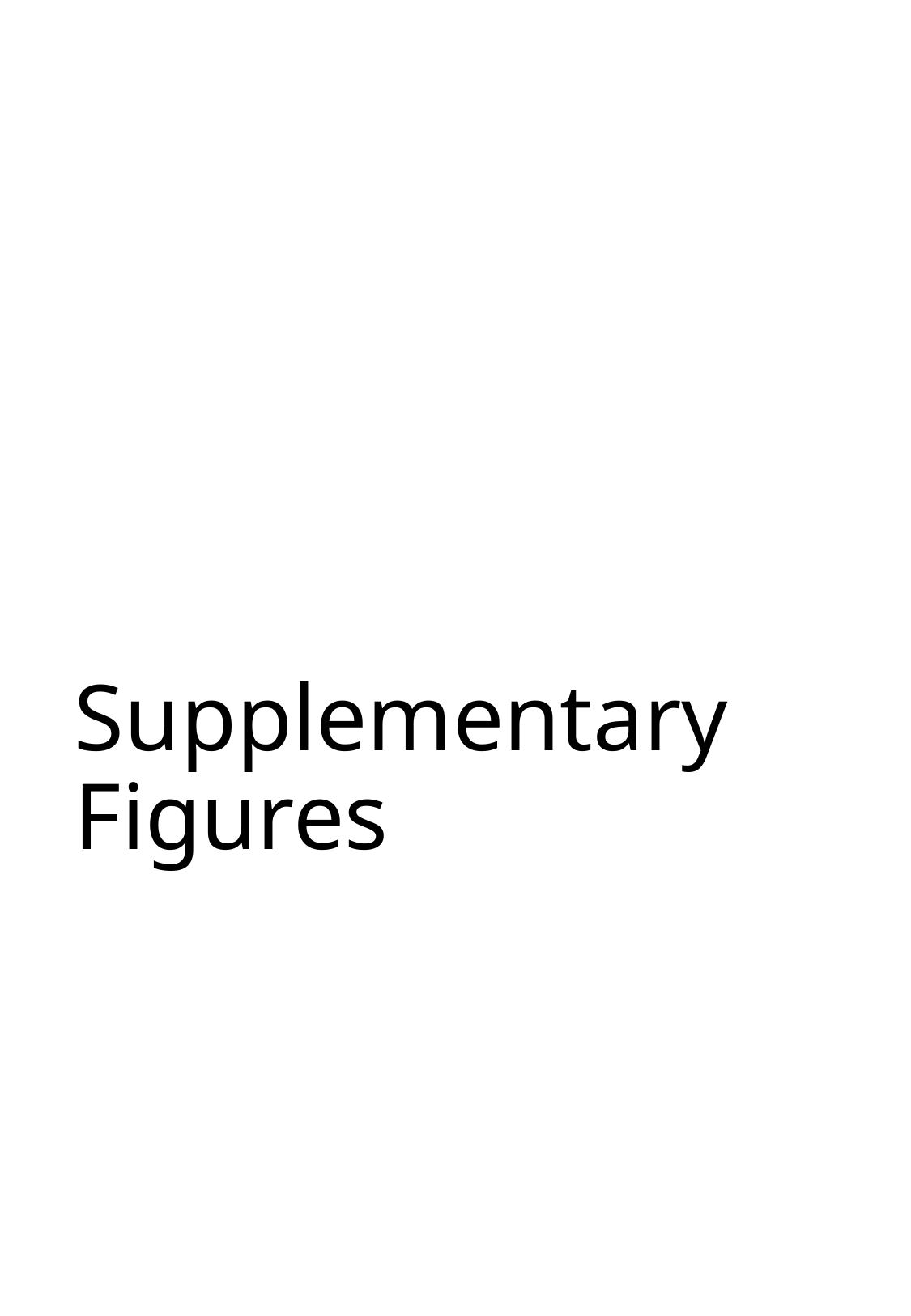

# Supplementary Figures

## Slide 2
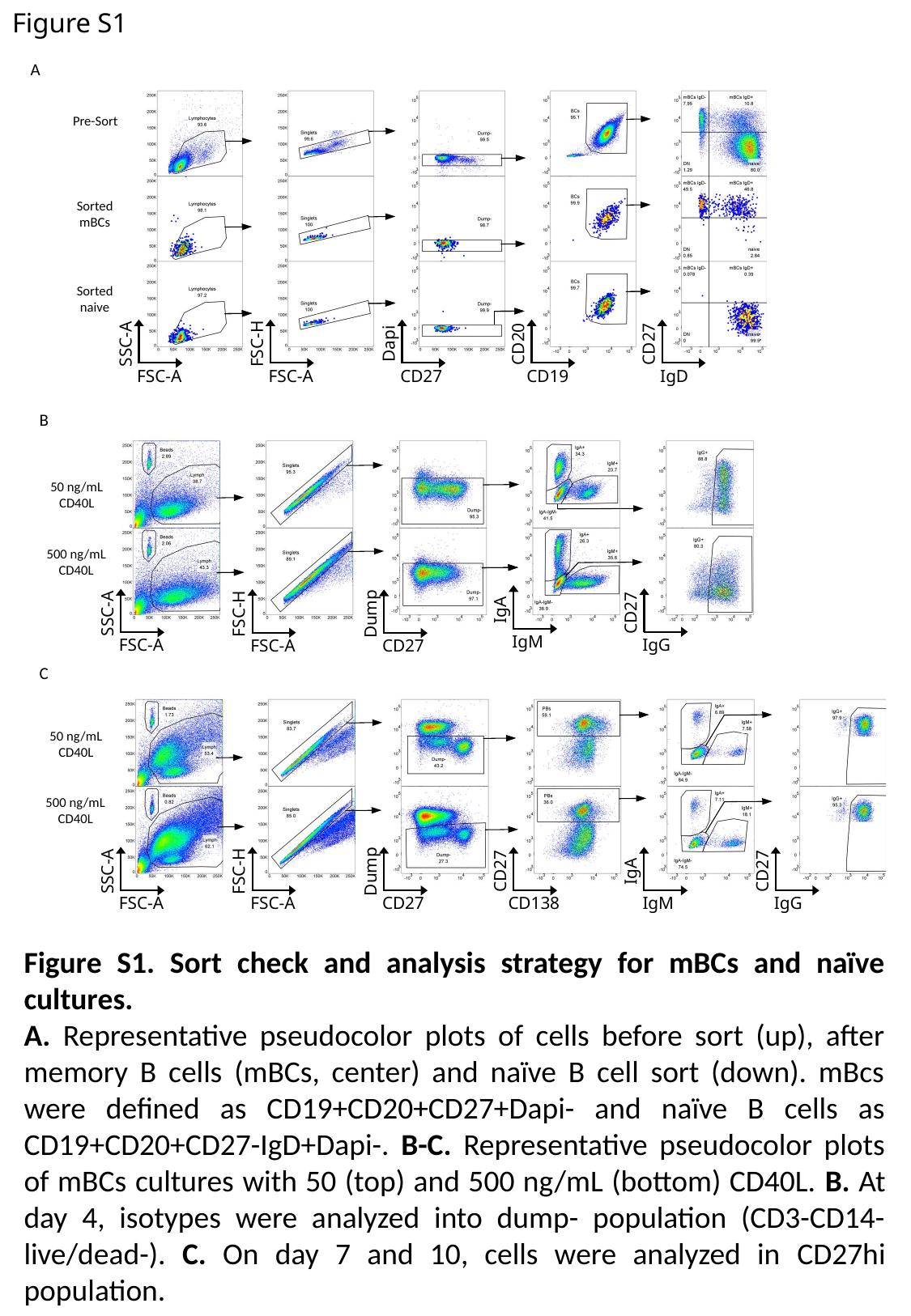

# Figure S1
A
Pre-Sort
Sorted
mBCs
Sorted
naive
FSC-H
FSC-A
SSC-A
FSC-A
CD20
CD19
CD27
IgD
Dapi
CD27
B
50 ng/mL CD40L
500 ng/mL CD40L
Dump
CD27
FSC-H
FSC-A
SSC-A
FSC-A
CD27
IgG
IgA
IgM
C
50 ng/mL CD40L
500 ng/mL CD40L
Dump
CD27
FSC-H
FSC-A
SSC-A
FSC-A
CD27
IgG
CD27
CD138
IgA
IgM
Figure S1. Sort check and analysis strategy for mBCs and naïve cultures.
A. Representative pseudocolor plots of cells before sort (up), after memory B cells (mBCs, center) and naïve B cell sort (down). mBcs were defined as CD19+CD20+CD27+Dapi- and naïve B cells as CD19+CD20+CD27-IgD+Dapi-. B-C. Representative pseudocolor plots of mBCs cultures with 50 (top) and 500 ng/mL (bottom) CD40L. B. At day 4, isotypes were analyzed into dump- population (CD3-CD14-live/dead-). C. On day 7 and 10, cells were analyzed in CD27hi population.

## Slide 3
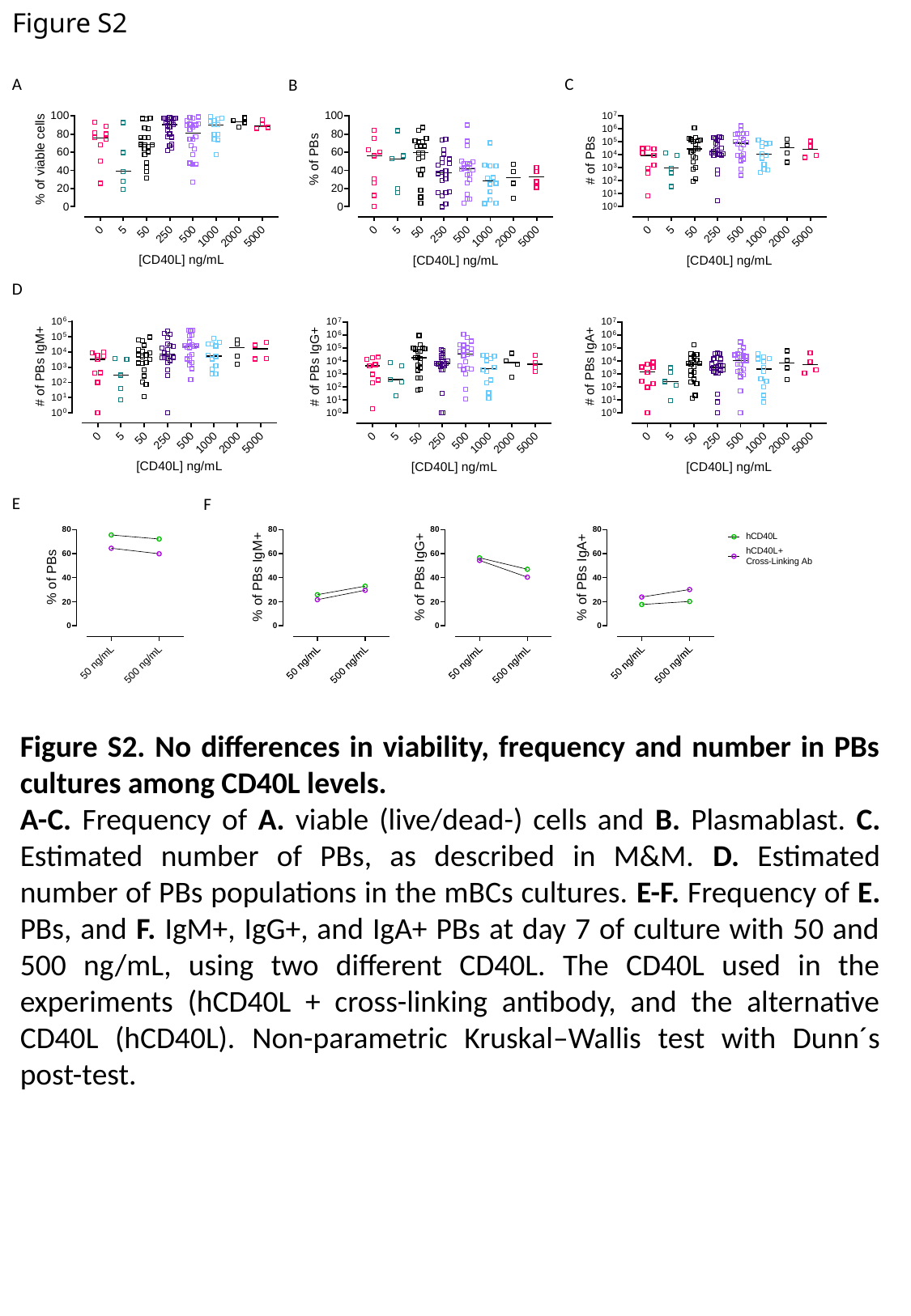

# Figure S2
C
A
B
D
E
F
Figure S2. No differences in viability, frequency and number in PBs cultures among CD40L levels.
A-C. Frequency of A. viable (live/dead-) cells and B. Plasmablast. C. Estimated number of PBs, as described in M&M. D. Estimated number of PBs populations in the mBCs cultures. E-F. Frequency of E. PBs, and F. IgM+, IgG+, and IgA+ PBs at day 7 of culture with 50 and 500 ng/mL, using two different CD40L. The CD40L used in the experiments (hCD40L + cross-linking antibody, and the alternative CD40L (hCD40L). Non-parametric Kruskal–Wallis test with Dunn´s post-test.

## Slide 4
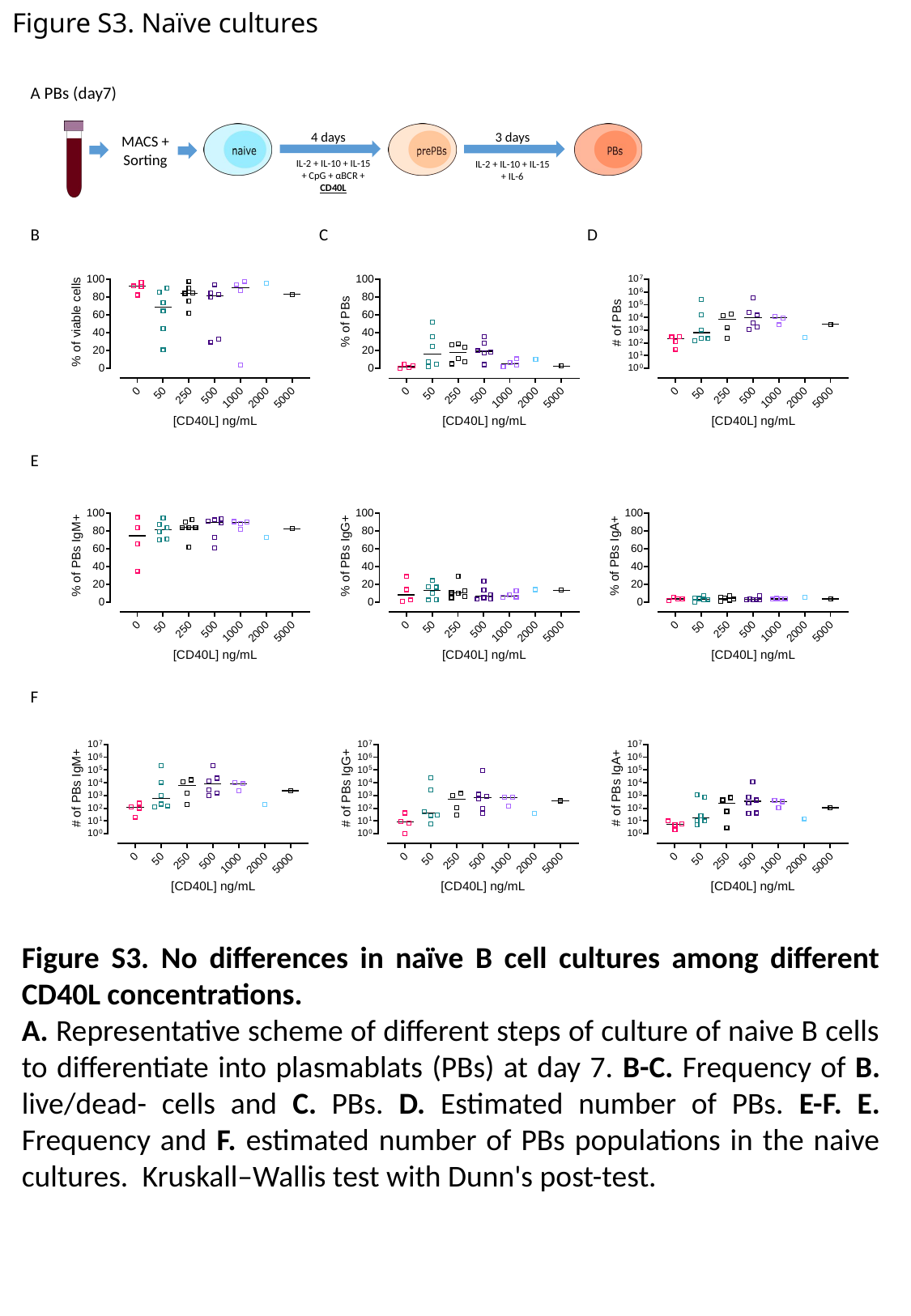

# Figure S3. Naïve cultures
A PBs (day7)
4 days
3 days
MACS +
Sorting
IL-2 + IL-10 + IL-15
+ CpG + αBCR +
CD40L
IL-2 + IL-10 + IL-15
+ IL-6
B
C
D
E
F
Figure S3. No differences in naïve B cell cultures among different CD40L concentrations.
A. Representative scheme of different steps of culture of naive B cells to differentiate into plasmablats (PBs) at day 7. B-C. Frequency of B. live/dead- cells and C. PBs. D. Estimated number of PBs. E-F. E. Frequency and F. estimated number of PBs populations in the naive cultures. Kruskall–Wallis test with Dunn's post-test.

## Slide 5
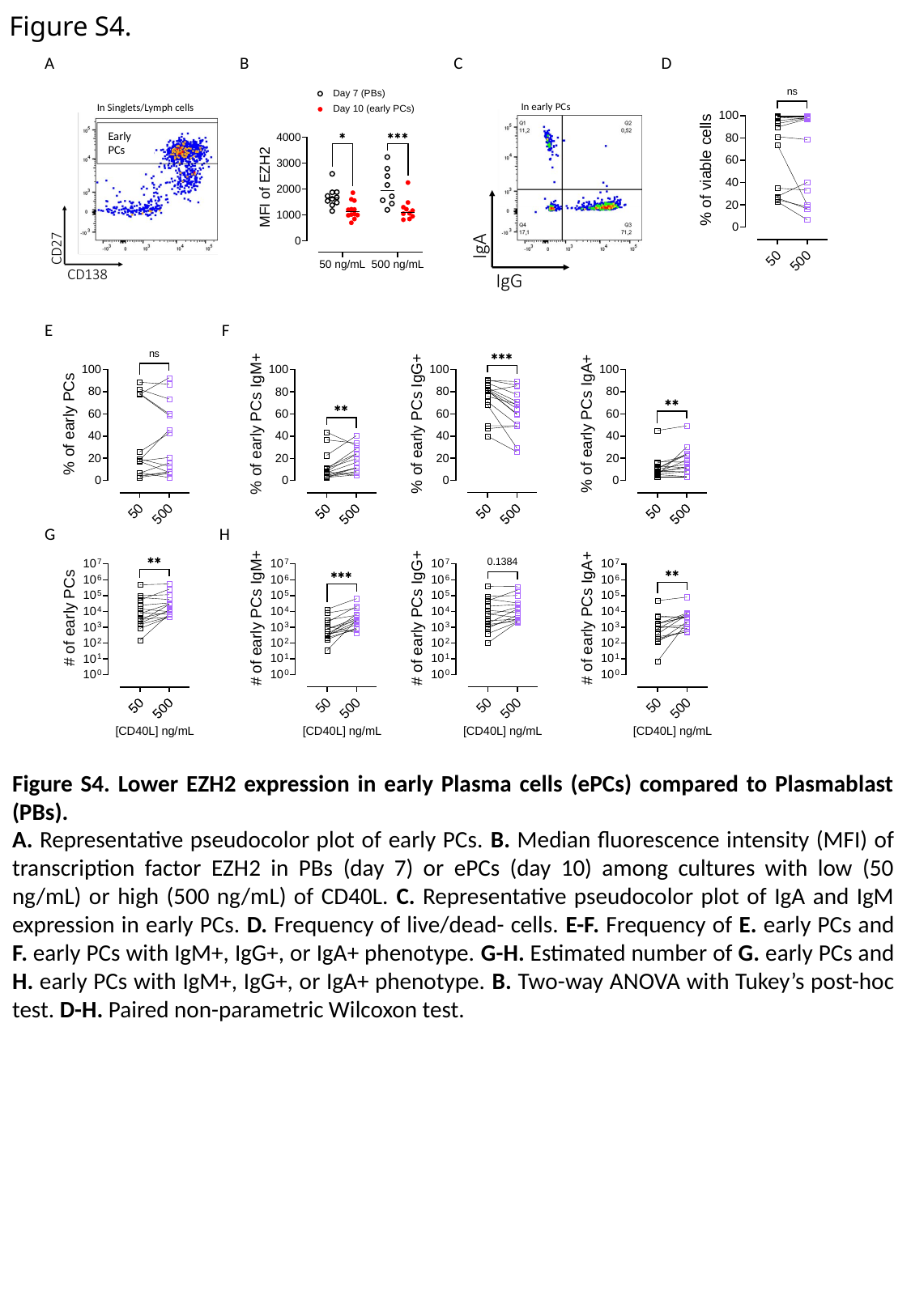

# Figure S4.
A
B
C
D
In early PCs
In Singlets/Lymph cells
Early PCs
E
F
G
H
Figure S4. Lower EZH2 expression in early Plasma cells (ePCs) compared to Plasmablast (PBs).
A. Representative pseudocolor plot of early PCs. B. Median fluorescence intensity (MFI) of transcription factor EZH2 in PBs (day 7) or ePCs (day 10) among cultures with low (50 ng/mL) or high (500 ng/mL) of CD40L. C. Representative pseudocolor plot of IgA and IgM expression in early PCs. D. Frequency of live/dead- cells. E-F. Frequency of E. early PCs and F. early PCs with IgM+, IgG+, or IgA+ phenotype. G-H. Estimated number of G. early PCs and H. early PCs with IgM+, IgG+, or IgA+ phenotype. B. Two-way ANOVA with Tukey’s post-hoc test. D-H. Paired non-parametric Wilcoxon test.

## Slide 6
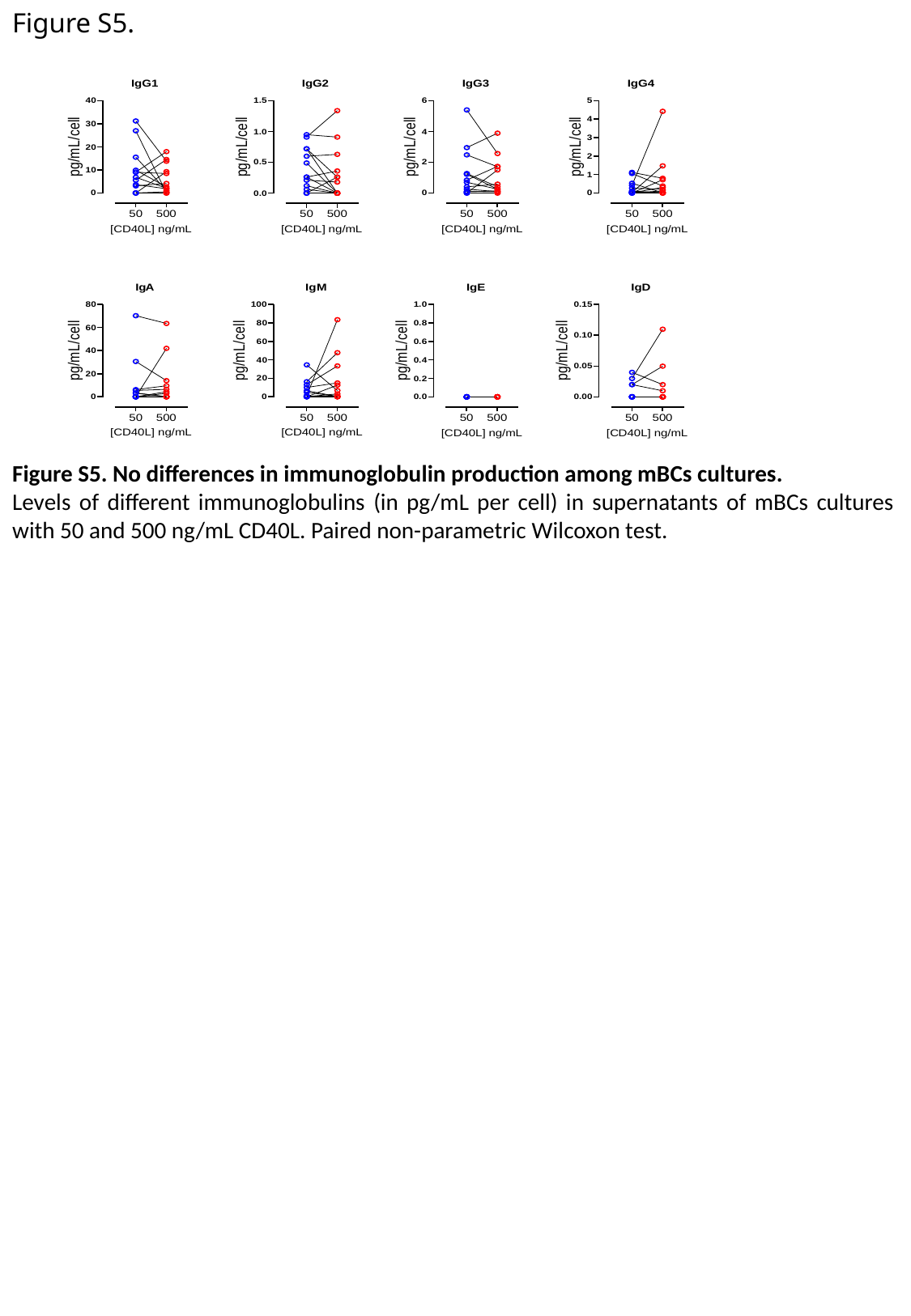

# Figure S5.
Figure S5. No differences in immunoglobulin production among mBCs cultures.
Levels of different immunoglobulins (in pg/mL per cell) in supernatants of mBCs cultures with 50 and 500 ng/mL CD40L. Paired non-parametric Wilcoxon test.

## Slide 7
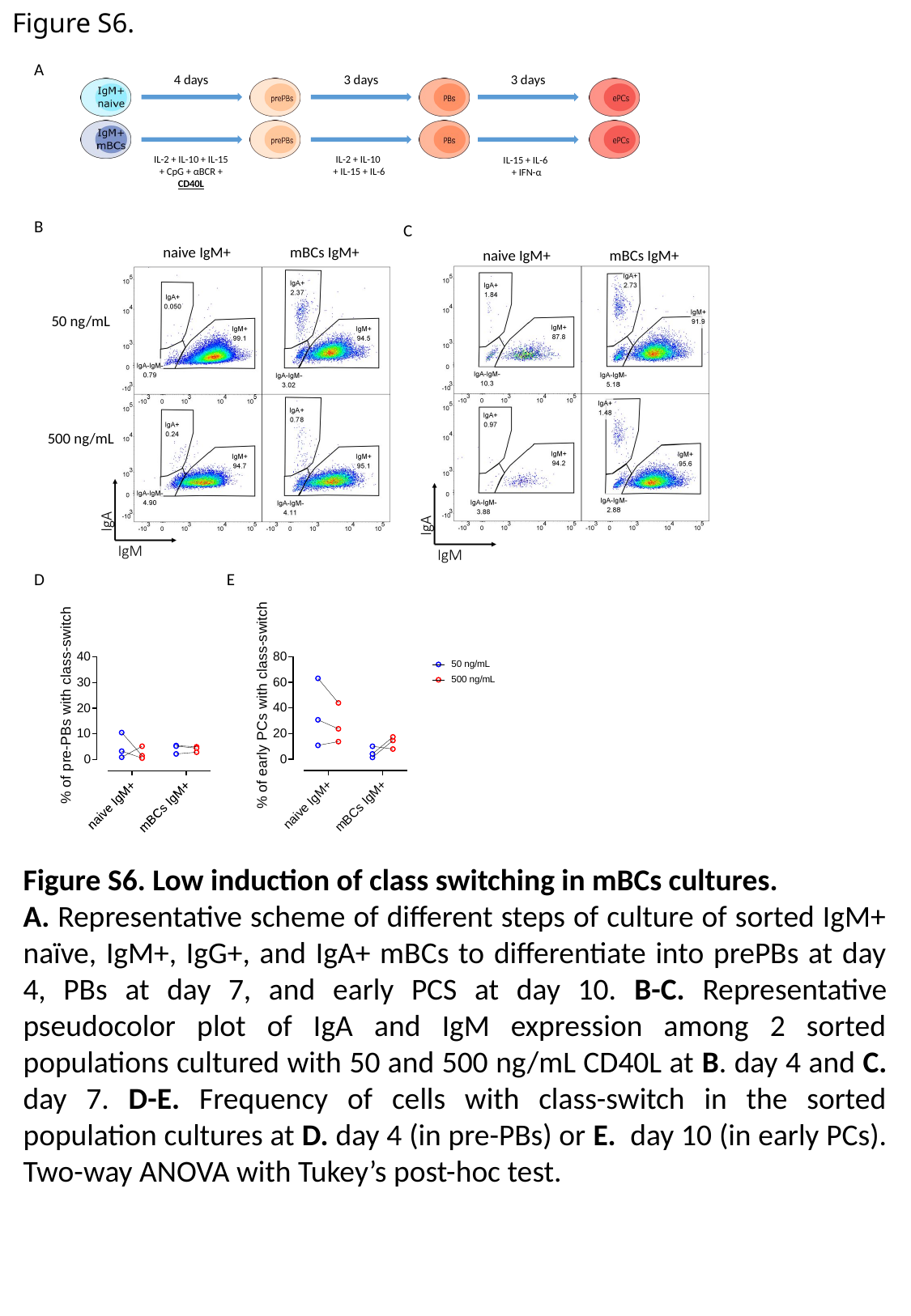

# Figure S6.
A
4 days
3 days
3 days
IL-2 + IL-10
+ IL-15 + IL-6
IL-2 + IL-10 + IL-15
+ CpG + αBCR +
CD40L
IL-15 + IL-6
+ IFN-α
B
C
naive IgM+
mBCs IgM+
naive IgM+
mBCs IgM+
50 ng/mL
500 ng/mL
D
E
Figure S6. Low induction of class switching in mBCs cultures.
A. Representative scheme of different steps of culture of sorted IgM+ naïve, IgM+, IgG+, and IgA+ mBCs to differentiate into prePBs at day 4, PBs at day 7, and early PCS at day 10. B-C. Representative pseudocolor plot of IgA and IgM expression among 2 sorted populations cultured with 50 and 500 ng/mL CD40L at B. day 4 and C. day 7. D-E. Frequency of cells with class-switch in the sorted population cultures at D. day 4 (in pre-PBs) or E. day 10 (in early PCs). Two-way ANOVA with Tukey’s post-hoc test.

## Slide 8
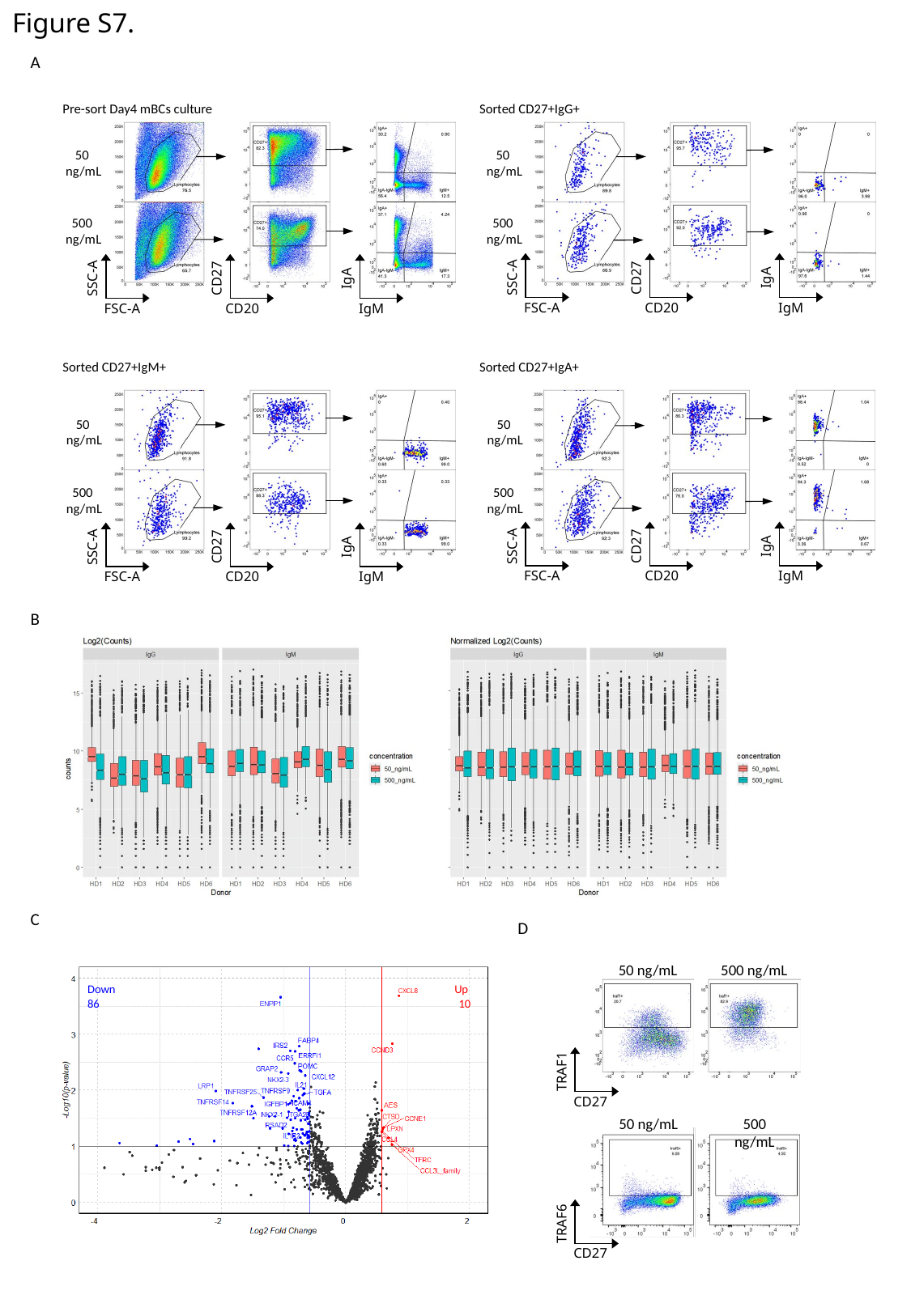

# Figure S7.
A
Pre-sort Day4 mBCs culture
Sorted CD27+IgG+
50
ng/mL
50
ng/mL
500
ng/mL
500
ng/mL
SSC-A
FSC-A
SSC-A
FSC-A
CD27
CD20
CD27
CD20
IgA
IgM
IgA
IgM
Sorted CD27+IgM+
Sorted CD27+IgA+
50
ng/mL
50
ng/mL
500
ng/mL
500
ng/mL
SSC-A
FSC-A
SSC-A
FSC-A
CD27
CD20
CD27
CD20
IgA
IgM
IgA
IgM
B
C
D
50 ng/mL
500 ng/mL
Down
86
Up
10
TRAF1
CD27
50 ng/mL
500 ng/mL
TRAF6
CD27

## Slide 9
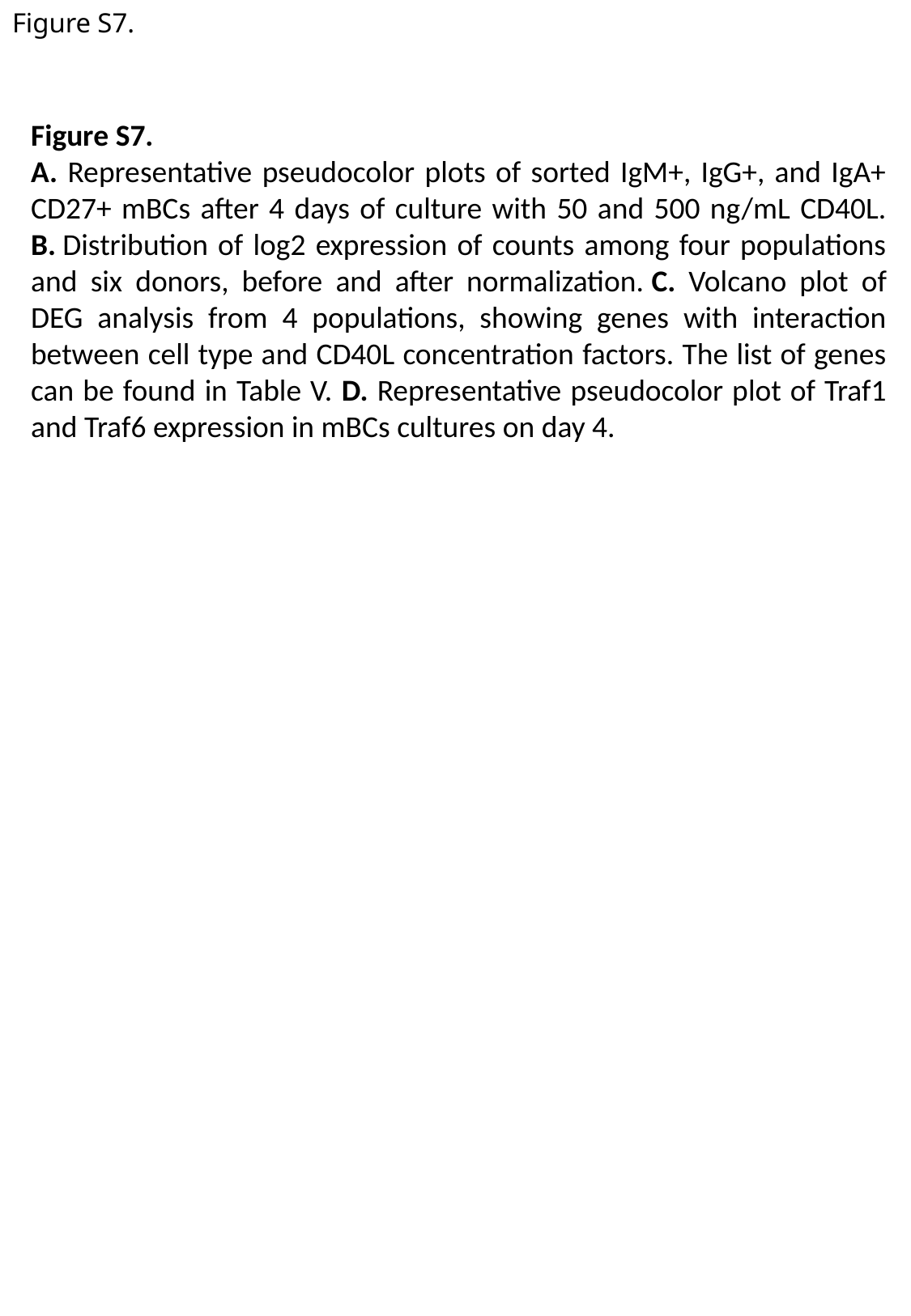

# Figure S7.
Figure S7.
A. Representative pseudocolor plots of sorted IgM+, IgG+, and IgA+ CD27+ mBCs after 4 days of culture with 50 and 500 ng/mL CD40L. B. Distribution of log2 expression of counts among four populations and six donors, before and after normalization. C. Volcano plot of DEG analysis from 4 populations, showing genes with interaction between cell type and CD40L concentration factors. The list of genes can be found in Table V. D. Representative pseudocolor plot of Traf1 and Traf6 expression in mBCs cultures on day 4.

## Slide 10
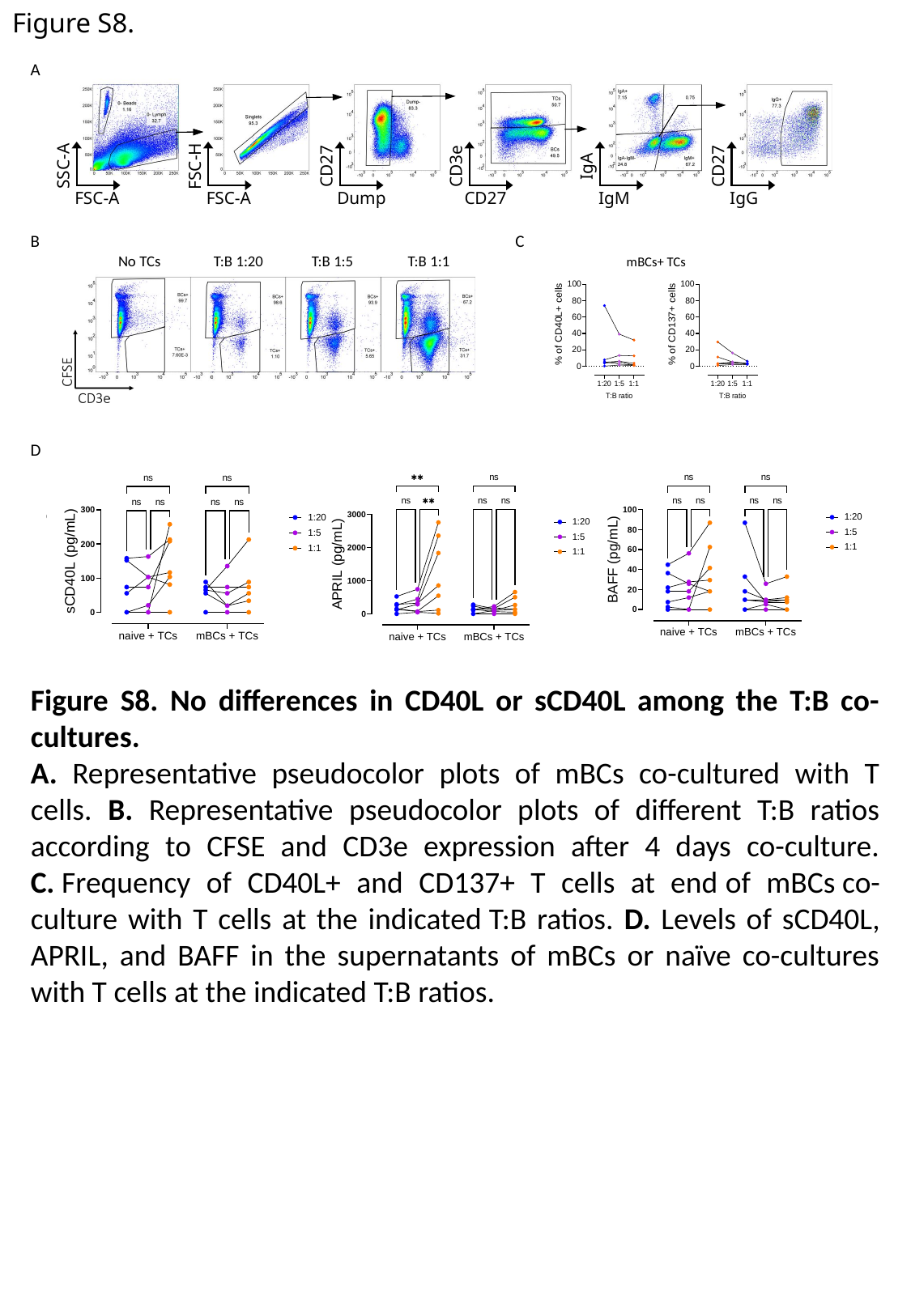

# Figure S8.
A
FSC-H
FSC-A
SSC-A
FSC-A
CD27
Dump
CD27
IgG
CD3e
CD27
IgA
IgM
B
C
No TCs
T:B 1:20
T:B 1:5
T:B 1:1
mBCs+ TCs
D
Figure S8. No differences in CD40L or sCD40L among the T:B co-cultures.
A. Representative pseudocolor plots of mBCs co-cultured with T cells. B. Representative pseudocolor plots of different T:B ratios according to CFSE and CD3e expression after 4 days co-culture. C. Frequency of CD40L+ and CD137+ T cells at end of mBCs co-culture with T cells at the indicated T:B ratios. D. Levels of sCD40L, APRIL, and BAFF in the supernatants of mBCs or naïve co-cultures with T cells at the indicated T:B ratios.
